# A Sub-Microanalysis Approach in Chemical Characterisation of Gold Nanorods Formed by a Novel Polymer-Immobilised Gold Seeds Base

**DOI:** 10.3390/nano7100331

**Published:** 2017-10-16

**Authors:** Majid Kazemian Abyaneh, Tohru Araki, Burkhard Kaulich

**Affiliations:** Diamond Light Source Ltd., Harwell Science and Innovation Campus, OX11 0DE Didcot, Oxfordshire, UK; tohru.araki@diamond.ac.uk (T.A.); burkhard.kaulich@diamond.ac.uk (B.K.)

**Keywords:** gold nanorods, PMMA, nanocomposites, SXM, NEXAFS, XRF

## Abstract

Gold nanorods (GNRs) have been fabricated by a novel polymer-immobilised seed mediated method using ultraviolet (UV) photoreduced gold-polymethylmethacrylate (Au–PMMA) nanocomposites as a seed platform and characterised at sub-micron scale regime with synchrotron-based techniques; near-edge X-ray absorption fine structure (NEXAFS) spectroscopy and X-ray fluorescence (XRF) mapping. In this report, it is shown that investigating polymer nanocomposites using combination of XRF mapping and NEXAFS spectromicroscopy can help to see the growth phenomenon from different perspective than conventional characterisation techniques. XRF maps are used to explore distribution of the constituent elements and showing how polymer matrix making stripe patterns along with regions where GNRs are formed. NEXAFS carbon (C) K-edge spectra have been taken at three different stages of synthesis: (1) on Au–PMMA nanocomposites before UV irradiation, (2) after gold nanoparticles formation, and (3) after GNRs formation. It reveals how polymer matrix has been degraded during GNRs formation and avoiding chemically or physically damage to polymer matrix is crucial to control the formation of GNRs.

## 1. Introduction

The past three decades have witnessed a tremendous growth of nanoscience, and a variety of methods have been proposed and developed to synthesize nanomaterials [1,2,3,4]. Undoubtedly, a fascinating aspect of nanomaterials is that their properties are size and shape dependent, which makes them attractive for science and industry and opens up new horizons for various novel applications e.g., biosensing [5], energy storage [6], catalysis [7], and advanced architectural materials [8]. One-dimensional nanomaterials have received intense attention, and novel applications for them have been found due to their interesting properties. Noble one-dimensional (1D) nanostructures, especially Gold Nanorods (GNRs), are important due to their unique optical and physical properties, which can find potential applications in optics, electronics, biology, etc. [9]. Many applications require growing GNRs on given surfaces that can be produced with either direct surface growth or indirect synthesis methods [10,11,12]. In direct methods, GNRs are physically or chemically grown directly on surfaces using pre-deposited seeds [13,14]. Indirect methods involve self-assembling pre-synthesised GNRs in which surfaces need to be chemically treated using various binders [15,16]. Each of these techniques is developed for a specific surface, which limit their application, and should be modified or redesigned for different surfaces. In this fast-growing science and technology field, there are always high demands for new and novel approaches to produce and fabricate one-dimensional nanomaterials that will definitely be of great interest to scientists and engineers, especially if the synthesis procedure is simpler and can be adopted with various surfaces and be used over extended areas and volumes. Recently, we proposed a proof of concept for formation of GNRs by a novel polymer-immobilised seed mediated method reported in reference [17]. For the first time, gold nanoparticles immobilised within PMMA polymer matrix were applied as seeds on which nanorods (NRs) grow using a seed-mediated method. The gold nanoparticles are formed with a UV photoreduction technique embedded in a polymer matrix to create an Au–PMMA nanocomposite. Metal-polymer nanocomposites are known as organic-inorganic hybrid materials within the larger research framework and have novel characteristics that combine the advantages of the integrated materials [18,19]. Polymers are able to immobilise metal nanoparticles, avoiding their agglomeration or segregation, thus protecting the novel size-dependent characteristics of nanoscale metals, which lead to fascinating functional materials. Our approach to synthesize GNRs on the surface of Au–PMMA nanocomposite films in solid form are of interest to many innovative fields where thin film coatings are applied like organic electronics [20,21]. The polymer-immobilised gold seeds layer can be formed over a wide range of substrates, and it is even possible to make freestanding films.

This work addresses a few missing elements in previous, works including control of the formation of GNRs in respect of their size and aspect ratio, while also studying and investigating the chemical composition and characterisation of the GNRs and base nanocomposites with suitable spectromicroscopy techniques like NEXAFS and XRF in a scanning X-ray microscope (SXM) beamline. For an extensive discussion on conventional characterisation techniques of gold nanomaterials, see a review article by A. Villa et al. [22].

## 2. Results and Discussion

### 2.1. Synthesised GNRs Using Polymer-Immobilised Seed Mediated Method

**Figure 1:**
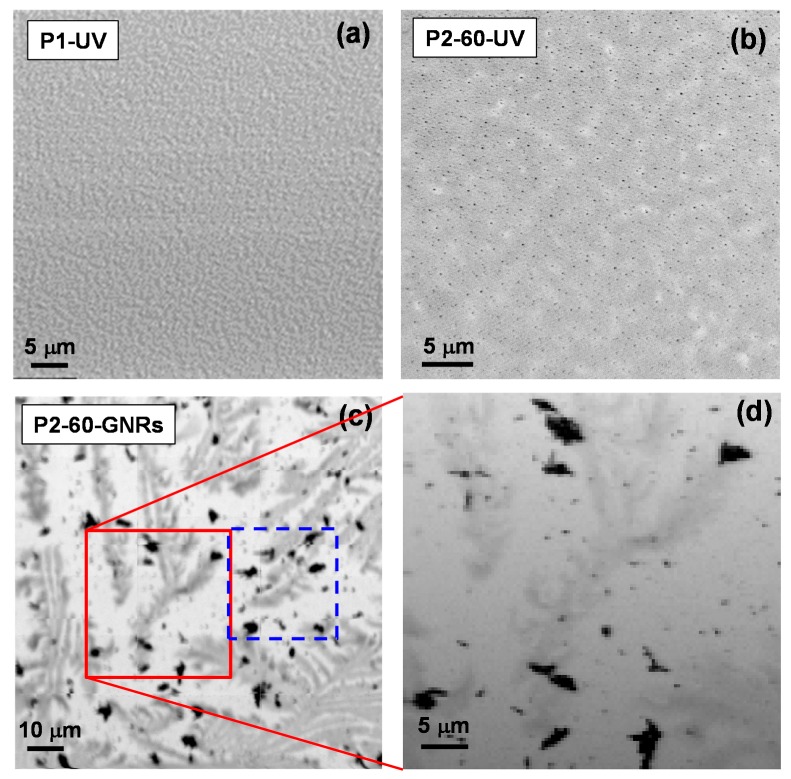
Scanning X-ray microscope (SXM) images for (**a**) low *M*w PMMA after UV irradiation; (**b**) Au–PMMA formed by UV irradiation and (**c**) gold nanorods formed by polymer immobilised seeds over Au–PMMA nanocomposite; image (**d**) is magnified image corresponding to the region assigned by red square in (**c**). The blue marked square is demonstrating a region of interest that has selected for X-ray fluorescence (XRF) mapping in Figure 3.

There are many reports demonstrating the formation of gold nanoparticles in a polymer matrix and also to produce well defined geometries of gold nanoparticles by UV photoreduction method [23,24]. It has been shown that by increasing gold salt concentrations, particle size decreases [25]. Protruded gold nanoparticles formed over the PMMA matrix provide a well-dispersed seed platform for the growth of GNRs. Figure 1 shows evolution of GNRs formation using a polymer-immobilised gold seeds method proposed recently for the first time by the author [17]. Figure 1a shows an X-ray absorption image in transmission scale (SXM image taken at *E_hν_* = 550 eV) of low molecular weight (*M*w) PMMA (P1) spin casted onto a silicon nitride membrane and irradiated by UV light (assigned as P1-UV) resulting in a speckle pattern. In an absorption image, any changes in grey scale can be caused by changes in topographic morphology of the sample. The absorption image is in compliance with the scanning electron microscope (SEM) and atomic force microscope (AFM) images taken on UV irradiated PMMA and reported in our previous publications [17,25]. Figure 1a represents surface roughening that is depicted as a result in variation of mechanical and chemical properties of polymer due to main chain scission [26,27]. Figure 1b shows X-ray absorption image (at *E_hν_* = 280 eV) of the formed gold nanoparticles over the higher *M*w PMMA (P2) matrix by UV irradiation (Assigned as P2-60-UV). The image is taken at energy below C K-edge absorption showing good contrast between PMMA and gold nanoparticles. As can be seen in Figure 1b, black spots representing Au nanoparticles are well distributed onto the surface of the polymer matrix appearing as a grey scale background. The average distance for the P2-60-UV nanocomposite is 180 nm with a narrow distribution of ±50 nm. The mean particle size for P2-60-UV sample is 50 nm with surface density of $3\times 10^{9}$ (nanoparticles/cm^2^). Size and shape dependence of Au–PMMA nanocomposites formed by UV irradiation are well documented by the authors [25]. In Figure 1b, one also can observe a different morphology with larger patches in grey scale changes than P1 in Figure 1a. These well-dispersed and polymer-immobilised gold nanoparticles formed on the surface of Au–PMMA nanocomposite have been used as a base platform onto which GNRs can be grown.

Figure 1c demonstrates how GNRs have been formed over surface of Au–PMMA nanocomposite with 60 wt % (assigned as P2-60-GNRs) using a novel polymer-immobilised gold seeds mediated method [17]. Figure 1d is a magnified image of the region in Figure 1c demarcated with a red squared frame. GNRs appear as black patches, which represent agglomerated rods randomly distributed all over the surface of P2-60-GNRs. The morphology of the polymer surface has been changed, and dendrite shapes have been developed. The formation of nanorods (NRs) associated with significant changes in the polymer matrix and this dynamic environment have an impact on the GNRs production. We have already shown how polymer molecular weight and the gold salt-to-polymer weight ratio can unprecedentedly affect the growth rate and distribution of GNRs [17]. It is concluded that photoreduced nanocomposite formed with a lower *M*w PMMA and 20 wt % gold salt provides suitable medium for growing well-dispersed GNRs with average dimension of 200 nm in length and aspect ratios up to 10.

Another overview of the Figure 1a–c shows that morphology of the composites undergoes huge deformation and changes by UV irradiation and later exposure to chemical solvents used in a seed-mediated route to form GNRs produces complicated surface changes. Surely, a better understanding of the mechanism involved in this dynamic medium will provide knowledge to tackle problems with tuning size and the shape of the nanostructures, which can be reached by new techniques to reveal their chemistry and elemental characteristics.

### 2.2. Elemental Mapping Using Soft X-Ray Fluorescence

**Figure 2:**
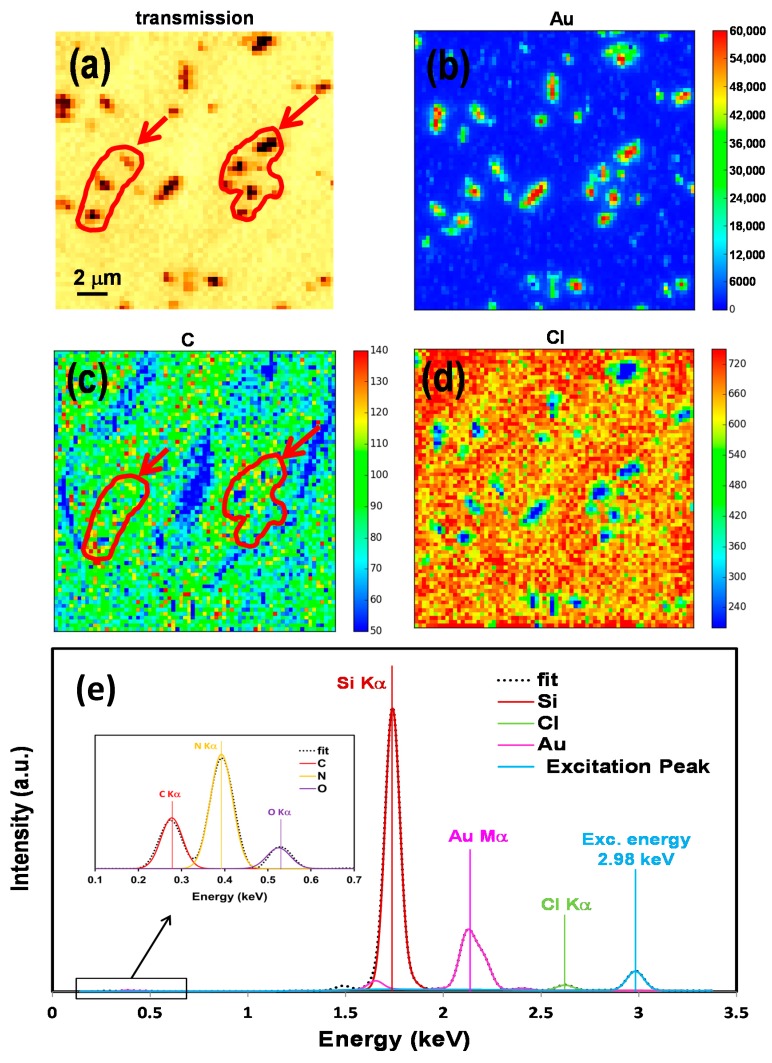
SXM image and XRF maps for P1-20-GNRs. (**a**) Transmission image taken at 2.98 keV; (**b**) XRF map for Au Mα; (**c**) XRF map for C Kα; (**d**) XRF map for Cl Kα; and (**e**) typical X-ray fluorescence spectrum from SDD detector fitted and processed using PyMCA software. Inset shows magnified region of low energy part of XRF spectrum including C Kα, N Kα, and O Kα signals. N Kα and Si Kα signals are from Si_3_N_4_ substrate. The two red contours, pointed out by arrows in carbon map (**c)** are drawn for clarification and showing the equivalent location of two chosen GNRs patches in image (**a**).

It also should be considered that any images taken by means of a scanning electron microscope (SEM) or atomic force microscope (AFM) provide evidence about the morphology of the sample but not the direct chemical information of the components. There are many techniques by which one can gain elemental and chemical information. Synchrotron-based X-ray fluorescence (XRF) and near edge X-ray absorption fine structure (NEXAFS) spectroscopy techniques are well established for elemental mapping and chemical speciation of materials, respectively. The combination of SXM data with XRF maps and NEXAFS spectra provide an insight into such a dynamic environment during the formation of GNRs using a polymer-immobilised gold seed–mediated method. We have used an X-ray fluorescence technique to acquire elemental mapping of the samples. SXM image and XRF maps for P1-20-GNRs are shown in Figure 2. Figure 2a shows the SXM image in transmission scale of the P1-20-GNRs that was acquired simultaneously with XRF data using a fibre optics–coupled CCD camera. Individual GNRs are formed in combination with agglomerated rods over the surface of polymer matrix. The excitation photon energy equal to 2.98 keV has been set to cover constituent edges in samples C Kα, O Kα, Au Mα, and Cl Kα.

X-ray fluorescence emission signal have been recorded pixel by pixel using a silicon drift detector (SDD) and analysed with PyMCA software [28] to create the elemental maps presented in Figure 2b–d. Figure 2b shows the gold elemental map (Au) where GNRs and gold nanoparticle distribution are bold with high counts. We have used a qualitative analysis where relative profound intensities of a given elemental edge apply to produce elemental maps. The high contrast between the blue and the other colour concludes that entire gold atoms are contributing to the formation of gold nanoparticles and NRs and gold materials are fully localized. Figure 2c presents the XRF map of carbon (C), which represents the polymer matrix. The carbon map shows a striped pattern on the surface, which has quite different morphology from the polymer pattern before formation of GNRs (Figure 1b). Note that such a pattern would not be recognized by transmission image alone, as polymer film is almost transparent for such high X-ray energies. The C stripe pattern suggests that the polymer is accumulating where GNRs are formed. To aid the reader, two red contours, pointed out by arrows, are drawn surrounding the GNRs patches for clarification. Figure 2d is devoted to the chlorine (Cl) map and shows chlorine distribution over the polymer matrix. It almost uniformly covers the polymer surface and is associated with the C pattern as well. It is proposed that AuCl_4_^−^ ions, which are sourced from gold salt, play a main role in forming passivation units for capping reduced gold atoms, and their concentration can control particles size and the nanoparticle population [25].

**Figure 3:**
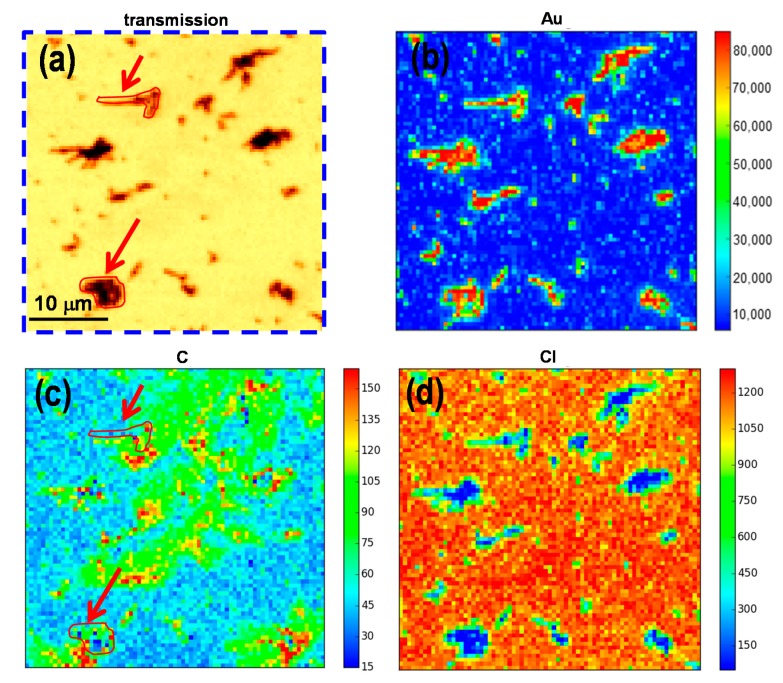
SXM image and XRF maps for P2-60-GNRs. (**a**) transmission image taken at 2.98 keV; (**b**) XRF map for Au Mα; (**c**) XRF map for C Kα; (**d**) XRF map for Cl Kα. The two red contours, pointed out by arrows in carbon map (**c**) are drawn for clarification and showing the equivalent location of two chosen GNRs patches in image (**a**).

A typical fluorescence spectrum from SDD detector is shown in Figure 2e and fitted using PyMCA software. Interesting peaks are the excitation peak at 2.98 keV, Cl Kα, Au Mα, and Si Kα peaks (from substrate), which are well separated and can be recognised, as shown in the graph. The inset shows the magnified part of the lower energy range, which is including C Kα, N Kα (from substrate), and O Kα peaks. For comparison, XRF data has been collected for P2-60-GNRs and is shown in Figure 3. Figure 3a shows the transmission image of the same blue square–marked region drew in Figure 1c. Combinations of individual GNRs and agglomerated rods can be observed to be irregularly distributed all over the surface of the sample. Larger rod patches are formed compared to the low *M*w polymer with lower gold salt concentration (P1-20-GNRs at Figure 2a). The XRF maps corresponding to Au, C, and Cl are presenting in Figure 3b–d, respectively. Figure 3b shows the GNRs patches and gold nanoparticles in red and yellow colour scales distributed on the surface of polymer matrix represented in background as blue. More particles, along with NRs, are observed on the surface compared to the P1-20-GNRs in Figure 3b. It seems that the particles that did not take part in the formation of GNRs have grown into larger particles with different shapes.

Figure 3c reveals that what we see as dendrites pattern in Figure 1c are actually accumulations of polymer, which are shown as green areas, and higher density of carbon, shown in red and yellow spots. In agreement with the P1-20-GNRs sample, we can see a higher density of carbon around the GNRs patches in sample P2-60-GNRs. Two of these patches are shown in Figure 3a,c, with red contours for clarification and pointed out with arrows. Figure 3d shows that chlorine is uniformly distributed over the polymer surface and does not resemble the carbon pattern, as in the case for the P1-20-GNRs (Figure 2d). X-ray fluorescence maps have shown that during the formation of GNRs, the polymer matrix evolved a lot, and such a dynamic environment can substantially affect growth parameters like the size and shape of GNRs. The polymer matrix in these samples acts as an immobiliser and holds the gold seeds in place over the surface. Any changes in the matrix will end up with the displacement of the Au seeds during the GNRs’ growth, which in turn will increase segregation and agglomeration of nanorods and consequently changes in size and shape of the GNRs.

### 2.3. Chemical Characterisation Using Soft X-Ray Spectromicroscopy

To gain a deeper insight, we have used NEXAFS spectromicroscopy at the I08-SXM beamline, to explore the chemical characterisation of the polymer matrix. A stack of SXM images is collected on a given energy range with defined steps around the absorption edge for the under test element. A stack of images can be aligned, normalized, and converted to optical density, from which NEXAFS spectra can be extracted pixel by pixel. The MANTiS software program (2nd Look Consulting, Hong Kong, China) has been employed for NEXAFS data analysis [29].

**Figure 4:**
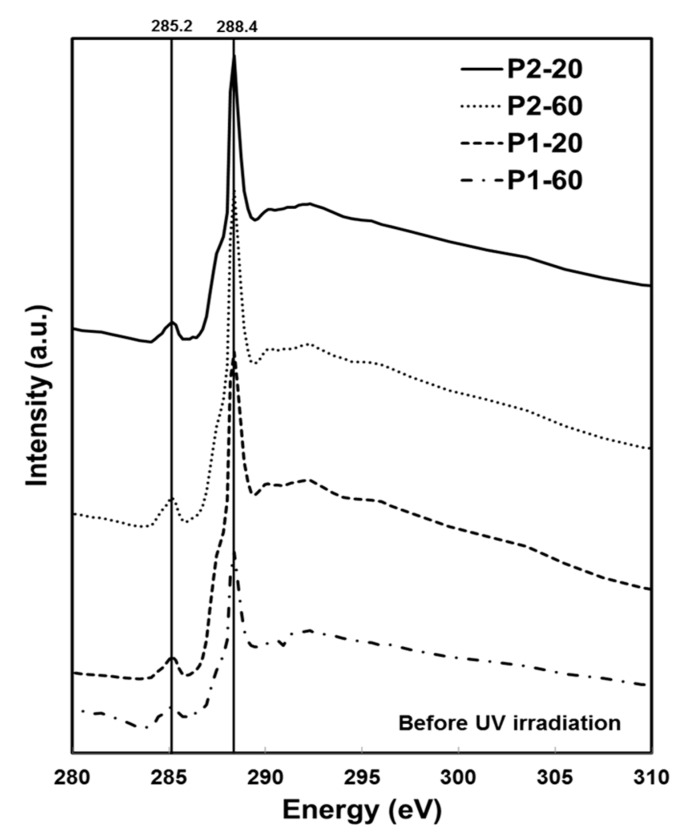
Near-edge X-ray absorption fine structure (NEXAFS) C1s K-edge spectra for P1-20, P1-60, P2-20, and P2-60 before UV irradiation.

**Figure 5:**
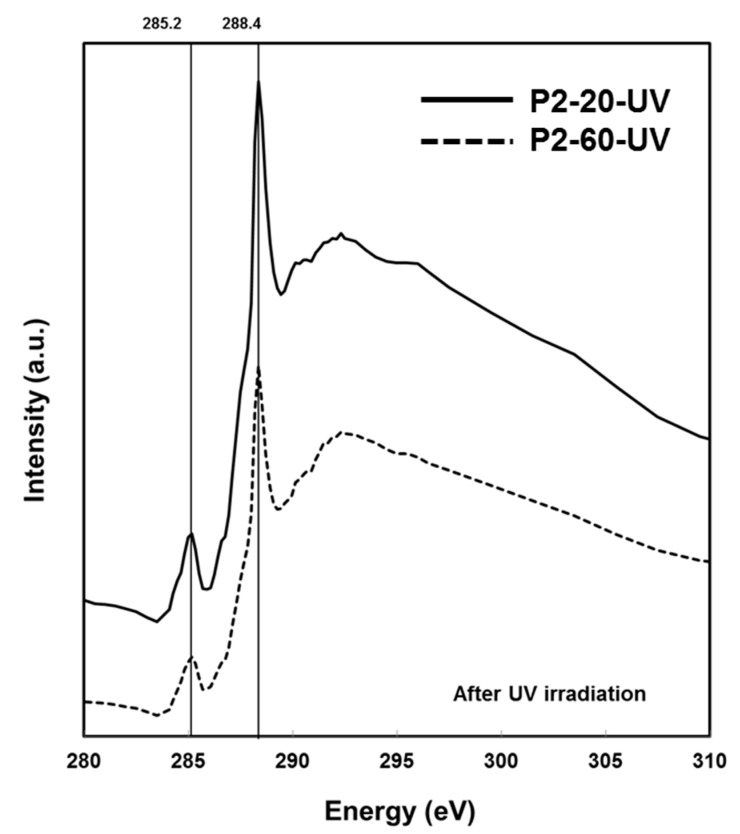
NEXAFS C1s K-edge spectra for P2-20-UV (solid line) and P2-60-UV (dash line) after UV irradiation.

NEXAFS spectra are extracted from images acquired at 0.1 eV energy intervals over the carbon K-edge absorption from 280–310eV in transmission mode. Absorption spectra of Au–PMMA samples before UV irradiation for low and high polymer molecular weight with different Au salt concentrations are shown in Figure 4. A characteristic peak at 288.4 eV can be observed that is known for C1s→π*_C=O_ transition in the carbonyl moiety of PMMA [30]. The other peak at 285.2 eV is for C1s(C=C)→π*_C=C_ transition, associated with unsaturated C=C bond. It is believed that later peak appears due to radiation damage on the polymer matrix. The most change in PMMA has been represented in a decrease of the 288.4 eV peak caused by C=O bond breaking, and the growth of the peak at 285.2 eV peak by forming C=C bonds simultaneously [31]. In a heavily damaged PMMA, the 288.4 eV peak disappears, and the 285.2 eV peak grows gradually. Having both peaks in Figure 4 evidences that the polymer matrix in Au–PMMA samples before irradiation is partially damaged regardless of polymer molecular weight and gold salt concentration, which could mainly be caused by SXM measurements. This can be mitigated by choosing a lower exposure dose [32]. Post-edge peaks at 290 eV and 296 eV are associated to C1s→σ*_C=O_ transition, which also disappear on damaged samples. Figure 5 demonstrates NEXAFS spectra for Au–PMMA nanocomposite samples, with high molecular weight and two different Au salt concentrations formed by UV irradiation method.

The characteristic peaks are similar to the peaks in Figure 4 at 285.2 eV and 288.4 eV. It is obvious that the peaks’ relative ratio has been changed, and the C1s(C=C)→π*_C=C_ signal at 285.2 eV is enhanced, while the C1s→π*_C=O_ signal at 288.4 eV is reduced. Part of this signal reduction is due to the cleavage of the ester group in PMMA.

**Figure 6:**
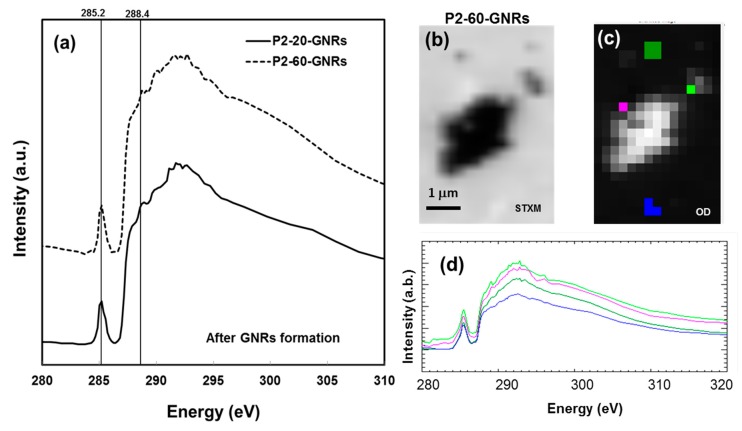
(**a**) NEXAFS C1s K-edge for P1-10-GNRs and P2-60-GNRs (after GNRs formation); (**b**) SXM image of a region of interest (ROI) in P2-60-GNRs sample chosen for detailed carbon spectroscopy; (**c**) Optical Density (OD) image of the chosen ROI with colour coded areas in which NEXAFS spectra have been extracted; (**d**) NEXAFS spectra corresponding to the coloured area shown in (**c**).

It is proposed that UV irradiation cleaves the carbonyl group from the backbone of the PMMA structure, which in turn plays a key role in the passivation of the Au nuclei forming gold nanoparticles in the PMMA polymer matrix [25]. The cleaved carbonyl group formed a passivating unit containing a weak bond with the Au atom, which in turn is used as a capping unit and is available for the passivation of photoreduced Au nuclei: in other words, preventing particle growth. There is no significant peak shift or change in the spectra shape at the pre- or post-edges, which emphasises that the effect of UV irradiation on polymer matrix is independent of the gold salt concentration. Figure 6a shows the C1s K-edge spectra of P2-20-GNRs and P2-60-GNRs assigned for GNRs formed on Au–PMMA nanocomposite with 20 wt % and 60 wt % ratio for higher molecular weight polymer, respectively. At first glance, it can be seen that the characteristic peak at 288.4 eV has disappeared, showing that the PMMA is heavily damaged. Along with that, the peak at 285.2 eV which is associated with the C1s(C=C)→π*_C=C_ signal, is sharply increased. The continuum signal at the post edge above 295 eV is visibly changed, which evidences a mass loss on the PMMA matrix [31].

At this stage, it is difficult to measure if mass loss differs in Au–PMMA samples with different molecular weights and gold salt concentrations, but it worth investigating by proper quantification like the one applied by Zhang et al. [33]. Figure 6b shows the SXM image of a region of interest (ROI) of agglomerated GNRs formed on P2-60-GNRs sample, in which detailed NEXAFS spectroscopy at sub-micron scale has been applied. The stacks of images like that shown in Figure 6b have been collected at energy steps over C1s K-edge, of which the NEXAFS spectra can be extracted and locally investigated. The stack can be normalized, and after dark subtraction, it forms an optical density image, which is shown in Figure 6c. Two regions at the neighbouring of nanorods (magenta and bright green) and two far from them (blue and dark green) have been chosen, and extracted C1s K-edge spectra corresponding to these regions are shown in Figure 6d. All regions are showing highly damaged polymer characteristics but regions closer to GNRs showing small carbonyl peak at 288.4 eV indicates less damage in regions closer to the formed GNRs.

**Figure 7:**
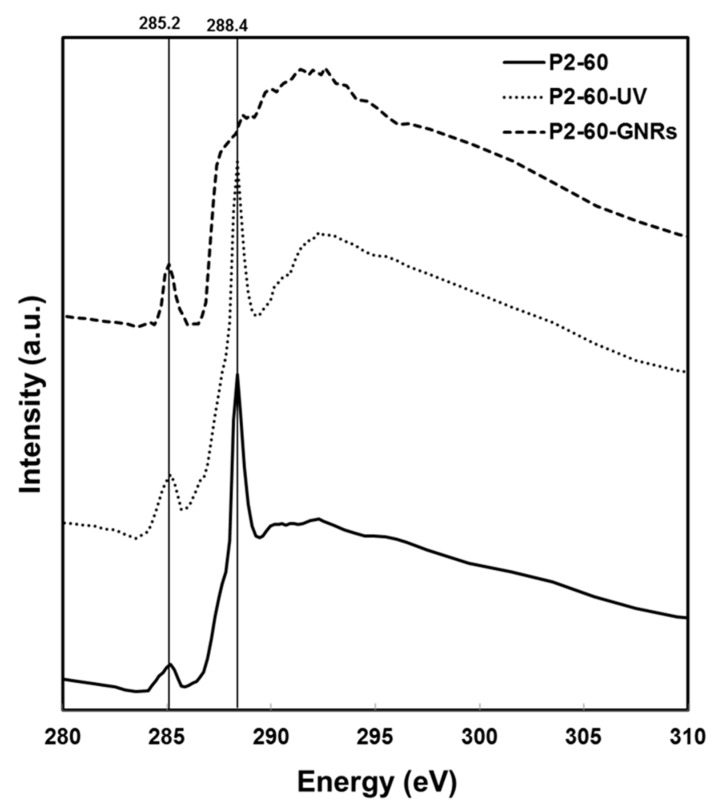
NEXAFS spectra of C1s K-edge for Au–PMMA with higher *M*w and 60% concentration in three stages of the GNRs formation, [P2-60] mixture of gold salt with PMMA polymer, [P2-60-UV] formation of Au–PMMA nanocomposite with 60 wt % gold using UV photoreduction, and [P2-60-GNRs] formed GNRs with seed mediated method over surface of P2-60 nanocomposite.

The post-edge peak’s shape associated to C1s→σ*_C=O_ transition is visibly different in the spectra for regions closer to GNRs than regions far from them. This is in agreement with the XRF data, which showed stripes morphology in C1s images of nearby regions of the GNRs (Figure 2c and Figure 3c). To have a better comparison, NEXAFS spectra of C1s K-edge of Au–PMMA with higher *M*w and 60% concentration in three stages of processing have been plotted in Figure 7.

It compares C1s K-edge spectra in three stages of the GNRs formation: (1) P2-60, mixture of gold salt with PMMA polymer; (2) P2-60-UV, formation of Au–PMMA nanocomposite with 60 wt % gold using UV photoreduction; and (3) P2-60-GNRs, formed GNRs with seed mediated method over the surface of P2-60 nanocomposite sample. It clearly shows that the signal at 285.2 eV grows, while the signal at 288.4 eV decreases and subsequently disappears on the last stage when the nanocomposite emerged in the growth solution to form GNRs. XRF and NEXAFS analysis reveal how the polymer matrix evolves during the formation of GNRs and exhibits how the polymer structure is less damaged nearby the GNRs and the stripe morphology shaped surrounding GNRs. This suggests that avoiding chemical or physical damage to the polymer matrix is the critical key to control the formation of GNRs using the polymer-immobilised gold seed–mediated method. The major change happened in stage 3, when nanocomposite film was immersed in the growth solution, and therefore the chemical interaction of the polymer matrix with the growth solution needs to be studied thoroughly. There are few parameters that need to be investigated that influence the polymer matrix. Among them, different solvents and stabilizing agents can be tested. Also, the effect of different physical parameters like immersion time, temperature, etc. should be considered. This is under investigation in our lab.

## 3. Conclusions

The elemental and chemical characterisation of gold nanorods formed by the polymer-immobilised gold seed–mediated method has been investigated using synchrotron-based XRF and NEXAFS techniques at the sub-micron scale. XRF maps show how constituent elements are distributed over the samples’ surfaces. The carbon XRF maps reveal a stripe pattern of the polymer matrix where the GNRs are formed, regardless of polymer molecular weight or gold salt concentrations. NEXAFS spectromicroscopy has been applied on the samples in three different stages: (1) on Au–PMMA nanocomposites before UV irradiation, (2) after gold nanoparticles formation, and (3) after GNRs formation. It is shown how the polymer structure has been changed after each of these stages. Changes in the characteristic peaks of the C spectra (carbonyl and graphitic peaks) determine how the polymer matrix has been degraded during GNRs formation, especially over the last stage, by immersing polymer-immobilised Au seeds in the growth solution. This leads us to conclude that avoiding chemical or physical damage to the polymer matrix is crucial to control the formation of GNRs by the polymer-immobilised gold seed–mediated method.

## 4. Materials and Methods

### 4.1. Synthesis of Au–PMMA Nanocomposite

Synthesis of Au–PMMA nanocomposites using UV irradiation has been reported previously in reference [25]. All materials and reagents were used as received. Briefly, 5 mL of acetone solution containing 0.5 g PMMA (*M*w = 120 kDa, density (*d*) = 1.188) was prepared in a glass tube and assigned as P1. Hydrogen tetrachloroaurate (III) trihydrate [HAuCl_4_-3H_2_O] was then added to 1 mL of the P1 solution to obtain 10%, 20%, 40%, and 60% weight ratio gold-polymer samples (assigned as P1-20; P1-40; P1-40; P1-60). The same procedure was followed to prepare P2-20 to P2-60 (P2 with *M*w = 996 kDa, *d* = 1.250). Subsequently, the final solutions were spin-casted on substrates (Si_3_N_4_ membrane with square window of 500 μm^2^ and 70 nm thickness). Finally, these films were exposed with a DC Deuterium (UV) 30 W lamp for 24 h.

### 4.2. Synthesis of Polymer-Immobilised GNRs

The experimental method for the formation of GNRs using the polymer-immobilised gold seed–mediated method has been thoroughly reported elsewhere [17]. Briefly, the Au–PMMA nanocomposites films were immersed into the 10 mL aqueous growth solution containing cetyltrimethylammonium bromide (CTAB) (100 mM), HAuCl_4_ (250 μM), ascorbic acid (500 μM), HNO_3_ (1 mM) and kept overnight at room temperature ~24 °C. After that, samples were thoroughly rinsed with DI water and left to dry in ambient conditions.

### 4.3. Synchrotron Based NEXAFS and XRF Measurements

**Figure 8:**
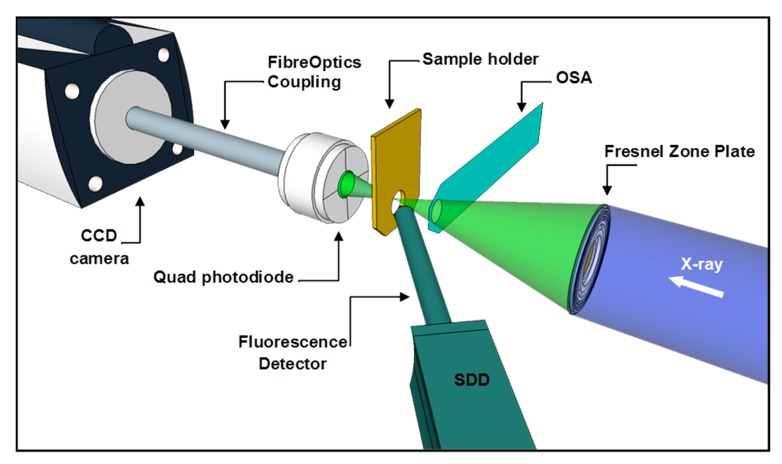
Schematic of SXM end-station at I08-SXM beamline. Other X-ray diffracted orders have not been shown for clarity.

C K-edge NEXAFS spectra and XRF measurements presented in this manuscript were carried out at the I08-SXM beamline at Diamond Light Source Ltd., Didcot, UK. The I08-SXM beamline hosts a scanning X-ray microscope (SXM) with multimodal detection capabilities. The I08-SXM uses radiation in the 250 to 4400 eV photon energy range, generated by an Apple II type undulator. This X-ray source is optimised to enable studies exploiting linearly or circularly polarised radiation. The operating energy range encompasses a significant number of important K and L absorption edges for low- and medium-Z elements, and relatively thick (~10–20 μm) samples can be studied with both absorption and phase contrast techniques, with lateral resolutions down to ~20 nm depending on the imaging mode. A schematic of the SXM end-station at the I08 is shown in Figure 8. Incoming X-rays are focused by means of a Fresnel zone plate, and undesired diffraction orders are eliminated by a pinhole called an order sorting aperture (OSA).

Sample raster scanning in focal plane and transmitted X-ray beam intensities are either directly measured by a photodiode (for NEXAFS data acquisition) or recorded on a CCD camera (Andor iXon Ultra, Andor Technology Ltd., Belfast, UK) through a fibre optic coupling configuration (for absorption and phase contrast imaging). Fluorescence-emitted signals are collected by a silicon drift detector (SDD) placed in front of sample plane at an angle of ~30°, which will be slide in close to the sample during the measurements (for XRF measurements).
